# Osteocytic Protein Expression Response to Doxercalciferol Therapy in Pediatric Dialysis Patients

**DOI:** 10.1371/journal.pone.0120856

**Published:** 2015-03-16

**Authors:** Renata C. Pereira, Harald Jüppner, Barbara Gales, Isidro B. Salusky, Katherine Wesseling-Perry

**Affiliations:** 1 Department of Pediatrics, David Geffen School of Medicine at UCLA, Los Angeles, California, United States of America; 2 Endocrine Unit and Pediatric Nephrology Unit, Massachusetts General Hospital and Harvard Medical School, Boston, Massachusetts, United States of America; Université de Lyon—Université Jean Monnet, FRANCE

## Abstract

**Background:**

Osteocytic protein expression is dysregulated in CKD and is affected by changes in mineral metabolism; however the effects of active vitamin D sterol therapy on osteocyte protein expression in advanced CKD is unknown.

**Methods:**

Eleven pediatric patients with end stage kidney disease underwent bone biopsy, were treated for 8 months with doxercalciferol, and then underwent a second bone biopsy. Bone expression of fibroblast growth factor 23 (FGF23), dentin matrix protein 1 (DMP1), and sclerostin were determined by immunohistochemistry and quantified by Ariol Scanning. Western blot analysis and qRT-PCR was performed on bone abstracts of a subset of study subjects to determine the nature (i.e. size) of FGF23 and DMP1 in bone before and after therapy.

**Results:**

As assessed by immunohistochemistry, bone FGF23, DMP1 and sclerostin protein all increased with therapy. In the case of FGF23, this increase was due to an increase in the full-length molecule without the appearance of FGF23 fragments. DMP1 was present primarily in its full-length form in healthy controls while 57kDa and 37kDa fragments of DMP1 were apparent in bone of dialysis patients at baseline and the 57 kDa appeared to decrease with therapy.

**Conclusion:**

Marked changes in osteocytic protein expression accompany doxercalciferol therapy, potentially impacting bone mineralization and the skeletal response to PTH. The effects of these bone changes on long-term outcomes remain to be determined.

## Introduction

Recent studies have identified that bone expression of fibroblast growth factor 23 (FGF23) increases early in the course of chronic kidney disease (CKD)[1] and is linked to abnormalities in skeletal mineralization, [2] redefining osteocytes as endocrine cells which generate hormones that affect both bone and the cardiovascular system.[2–4] PTH stimulates FGF23 production via activation of the nuclear receptor related 1 protein (Nurr1) [5] and potentially also via suppression of sclerostin;[6] however, increased expression of FGF23 in CKD occurs prior to detectable changes in circulating PTH concentration.[1, 2] Moreover, skeletal sclerostin expression increases in early CKD despite normal serum PTH levels[7] and continues to be increased even in end-stage kidney disease, despite elevated circulating PTH values.[8] Other data from humans and animals with genetic forms of hypophosphatemic rickets and normal kidney function have identified that dentin matrix protein 1 (DMP1), a member of the family of small integrin-binding ligand, N-linked glycoprotein (SIBLING) proteins, suppresses skeletal FGF23 expression.[9] In patients with early CKD, however, increased osteocytic FGF23 expression occurs in the face of increased osteocytic DMP1 expression.[2] Thus, the initial triggers for increased bone FGF23 expression in CKD remain unclear.

FGF23 processing and osteocyte biology appear to change with a progressive decline in kidney function. Although circulating FGF23 undergoes cleavage in patients with normal kidney function and in those with mild CKD,[10] the majority of circulating FGF23 in dialysis patients is in its full-length form [11] and changes in circulating mineral ion and hormone concentrations may play a significant role in osteocytic protein expression as CKD advances. Current data suggest that increasing PTH levels suppress osteocytic sclerostin expression;[12] however, bone sclerostin is increased in CKD.[7] Circulating phosphorus and PTH both increase FGF23 concentrations[13, 14] and therapies used to treat renal osteodystrophy, in addition to controlling secondary hyperparathyroidism and bone turnover, likely also alter osteocytic protein expression, potentially via changes in mineral metabolism. Indeed, vitamin D sterol therapy suppresses PTH while increasing circulating FGF23 levels dramatically in patients with end-stage kidney disease;[13] however, the effect of this form of therapy on skeletal expression of FGF23, sclerostin, and DMP1 in the context of advanced kidney disease and secondary hyperparathyroidism remains unknown. Thus, in order to assess the interplay between these three skeletal proteins and parameters of mineral metabolism during active vitamin D sterol therapy in advanced CKD, the current study evaluated iliac crest bone tissue in pediatric dialysis patients before and after 8 months of therapy with 1α-(OH)vitamin D_2_ (doxercalciferol).

## Materials and Methods

### Study Subjects

Eleven pediatric patients with end-stage kidney disease (ESKD) treated with peritoneal dialysis who had bone biopsies before and 8 months after the initiation of doxercalciferol therapy were candidates for the study. Active vitamin D sterol therapy was withheld during the 4 weeks preceding the initial bone biopsy [13] and high turnover renal osteodystrophy, as defined by an increase in bone formation rate and/or the presence of marrow fibrosis on the initial biopsy, was a pre-requisite for study participation. Exclusion criteria included: previous history of poor medication compliance; parathyroidectomy within the past 12 months; concurrent treatment immunosuppressive agents or growth hormone; or bone pathology not related to secondary hyperparathyroidism.

After the initial bone biopsy, subjects were treated for 8 months with a calcium-free phosphate binder (sevelamer carbonate), titrated to maintain serum phosphorus between 4.0 and 6.0 mg/dl [10;16], and an active vitamin D sterol (doxercalciferol). Doxercalciferol doses were initiated at 5.0 mcg/dose (15.0 mcg per week) and were adjusted during the study by the treating physician to achieve a target PTH level between 300 and 400 pg/ml while maintaining serum calcium levels between 8.4 to 10.2 mg/dl and phosphorus between 4 and 6 mg/dl. This target range was chosen based on a previous clinical trial in which a target of 300 to 400 pg/ml, in combination with normal serum calcium and phosphorus concentrations, resulted in normal rates of bone formation, without inducing adynamic bone disease, in the majority of pediatric patients treated with thrice weekly vitamin D analogues and phosphate binders.[13] Circulating values of calcium, phosphorus, and alkaline phosphatase values were assessed using an Olympus AU5400 analyzer (Olympus America Incorporated, Center Valley, PA). PTH concentrations in EDTA plasma were measured by the 1^st^ generation immunometric assay (Immutopics, San Clemente, California, normal range: 10–65 pg/ml) and FGF23 levels were determined in EDTA plasma by a 2^nd^ generation C-terminal assay (Immutopics, San Clemente, California). 25(OH)vitamin D values were measured by radioimmunoassay. [15] All biochemical values were assessed at baseline and at the time of the final bone biopsy. Circulating 1,25(OH)_2_vitamin D levels were measured at baseline and in the middle (month 3 to 4) of therapy to monitor medication compliance.

All subjects were treated with peritoneal dialysis; the dialysate calcium concentration was 2.5 mEq/l and the dextrose concentration was titrated to meet ultrafiltration goals. No patients were treated with growth hormone or immunosuppressive agents within the previous 6 months and none had undergone parathyroidectomy within the preceding year. Patients were removed from the study per protocol in the event of renal transplantation, medication noncompliance or change in dialytic modality.

### Ethics Statement

The study was approved by the UCLA Human Subject Protection Committee and written informed consent was obtained from all patients and/or parents.

### Bone Biopsy and Histomorphometry

Patients were admitted to the UCLA Clinical Translational Research Center and full thickness bone biopsies were obtained from the anterior iliac crest using a modified Bordier trephine (0.5 cm diameter) needle after double tetracycline-labeling.[16] A second smaller core (6 mm) was obtained with a Jamshidi needle and placed immediately in TRIzol (Invitrogen, Carlsbad, CA) for subsequent protein and RNA extraction. Specimens were fixed in 70% ethanol, dehydrated in alcohol, cleared with xylene, and embedded in methylmethacrylate. Static histomorphometric parameters were evaluated in undecalcified 5 μm sections stained with Toluidine blue; tetracycline labeling was assessed in unstained 10 μm sections.

Primary bone histomorphometric parameters were assessed in trabecular bone under 20x magnification using the OsteoMetrics system (OsteoMetrics, Decatur, GA) and classified under the Turnover, Mineralization and Volume (TMV) system.[17] Normal values were previously obtained from double-tetracycline labeled iliac crest specimens from 31 pediatric patients with normal kidney function undergoing elective orthopedic surgery.[16]

### Immunohistochemistry and Quantification of Bone FGF23, DMP1, and Sclerostin Expression

The technique for immunohistochemical detection of protein in bone was adapted from a previously reported method.[18] In brief, 5 μm sections of bone tissue were de-plastified in xylene and chloroform, rehydrated in graded alcohol solutions, and partially decalcified in 1% acetic acid. Endogenous peroxidase activity was quenched in 3% hydrogen peroxide/methanol solution. Non-specific binding was blocked in avidin-biotin solution and in 5% normal horse serum with 1% bovine serum albumin. Sections were incubated with affinity purified polyclonal goat anti-human FGF23(225–244) (Immutopics Intl, San Clemente, California) (dilution 1:500), monoclonal anti-human DMP1 (LFMb31)(62–513) (Santa Cruz Biotechnology, Inc., Santa Cruz, CA) (dilution 1:50), or monoclonal anti-human sclerostin (R&D Systems, Minneapolis, MN) (dilution 1:500) primary antibody overnight at 4°C in a humidified chamber. Sections were then incubated with biotinylated anti-goat (Vector, Burlingame, CA, USA), anti-mouse (Sigma-Aldrich, St. Louis, MO), or anti-rabbit (Sigma-Aldrich, St. Louis, MO) antibody; incubated for 30 minutes with StreptABC Complex/HRP kit (Vector) follow by AEC substract Chromogen (Dako, Carpinteria, CA); and counterstained with Mayer hematoxilin (Sigma-Aldrich).

Iliac crest bone biopsy specimens from 5 adolescent and young adult subjects with normal renal function comprised the “healthy control” population. Negative controls were performed for each bone section by omitting the primary antibody. Reproducibility was insured by repeating the immunohistochemistical analysis on all specimens. FGF23 and DMP1 were assessed in the entire area of trabecular bone and sclerostin was assessed in both cortical and trabecular bone. Immunoreactivity was quantified using the Ariol scanning system. All slides were scanned at 20x magnification with a red filter and digitized (Applied Imaging Inc., San Jose, CA). Analyzed fields were manually selected to avoid areas with tissue damage occurring during immunostaining. Staining was expressed as pixels/mm^2^.[19–22] All analyses were performed with the MultiStain script.

### Western Blot Analysis of Human Osteocyte Proteins

Fresh bone fragments of iliac crest measuring 6 mm in diameter were obtained from the bone biopsy procedure (see above). In order to avoid contamination from muscle and connective tissue, external edges of each core were removed prior to processing. Samples were pulverized in TRIzol (Invitrogen) and protein, RNA, and DNA were extracted according to the manufacturer’s instructions. The protein pellet was resuspended in 10% SDS and quantified using the DC Protein Assay Kit (BioRad, Hercules, CA). Ten micrograms of protein per sample were subjected to electrophoresis through a 15% SDS-polyacrylamide gel under reducing conditions and transferred to a PVDF membrane (Millipore, Billerica, MA). Membranes were blocked for 1 hour with 3% BSA in PBS and probed with affinity purified goat anti-human FGF23(224–255) antibody (dilution: 1:1000) (Immutopics, Int. San Clemente, CA), mouse anti-human DMP1 (LFMb31, Santa Cruz Biotechnology, Inc, Santa Cruz, CA) (dilution: 1:500), or rabbit anti-human beta-actin (dilution: 1:1000) (Abcam, Cambridge, UK) overnight. Blots were incubated for 2 hours with anti-goat or anti-mouse horseradish peroxidase (HRP) conjugated secondary antibody (Sigma-Aldrich, St. Louis, MO; 1:3000). Protein bands were visualized by SuperSignal West Pico chemiluminescence detection reagent (Thermo Scientific, Hudson, NH) and bands were visualized by fluororadiography on Reflection X-ray film (Thermo Scientific, Hudson, NH). Band intensity was quantified using ImageJ downloadable software (Softonic) and band intensity was normalized by ß-actin intensity. Intensity of each sample band was then divided by the intensity of the normalized control sample. Western blot experiments were performed at least twice. Membranes were stripped between the antibodies by using Restore PLUS Western BLOT stripping Buffer (Thermo Scientific, Hudson, NH).

### qRT-PCR

RNA was isolated from bone using TRIzol (Invitrogen), quantified spectroscopically, and treated with RQ1 DNase I (Promega). Real-time reverse transcription PCR (qRT-PCR) was performed and relative changes in gene expression using TaqMan primers (DMP1(Hs00189368_m1), phosphate regulating endopeptidase homolog, X-linked (PHEX) (Hs01011692_m1), FGF23 (Hs00221003_m1), bone morphogenic protein 1 (BMP1)(Hs 00241807_m1), and matrix metallopeptidase 2 (MMP2)(Hs01548727_m1) were calculated in relation to glyceraldehyde 3-phosphate dehydrogenase (GAPDH) levels using the 2^−ΔΔC(T)^ method.[23] In brief, the number of cycles required for target gene copy number to cross SYBR absorbance threshold (Ct) in each patient sample was subtracted from the number of cycles required to cross Ct in a normal control sample (ΔCt target). Similarly, the number of cycles required for GAPDH copy number to cross SYBR absorbance threshold (Ct) in each patient sample was subtracted from the number of cycles required to cross Ct in a normal control sample (ΔCt GAPDH). The overall increase in gene expression from normal control was calculated as 2^(ΔCt target)/2^ (ΔCt GAPDH). All samples were run in triplicate and cycle numbers represented the average of the three.

### Statistical Analysis

Measurements for normally distributed variables are reported as mean + standard error (SE); median values and interquartile range are used to describe non-normally distributed variables. Changes from baseline were assessed using the Wilcoxon signed-rank test and correlations evaluated according to Spearman coefficients. All statistical analyses were performed using SAS software (SAS Institute Inc., Cary, NC) and all tests were two-sided. A probability of type I error less than 5% was considered statistically significant and ordinary *p* values are reported.

## Results

### Patient Demographic Data, Biochemical Values, and Bone Histomorphometry at Baseline and After 8 Months of Therapy

Eleven pediatric dialysis patients (7 male and 4 female/ 9 Hispanic, 1 white, and 1 black) met entry criteria, agreed to participate, and were included in the study. The average age was 15.8 + 0.8 years and BMI was 21.7 + 2.2 kg/m^2^. Subjects had been on dialysis for an average of 1.1 + 0.4 years. All of the study participants had some residual urine output. During the course of therapy, subjects received an average of 19.3 + 3.8 mcg of doxercalciferol per week. Calcitriol levels increased by 10.9 + 5.0 pg/ml and doxercalciferol doses correlated with the change in circulating calcitriol concentration (r = 0.55, p = 0.08). Baseline and final biochemical parameters are shown in **Table 1**. At baseline, serum calcium levels were 8.6 + 0.2 mg/dl and serum phosphorus concentrations were 6.3 + 0.4 mg/dl; neither parameter changed with doxercalciferol therapy. Plasma PTH, alkaline phosphatase, and FGF23 levels were elevated at baseline. Overall, 6 patients achieved PTH levels within the target range while values remained elevated in the remainder due to the development of hypercalcemia and hyperphosphatemia. While PTH and alkaline phosphatase values did not change with therapy, FGF23 concentrations increased and final values were 1696 (565, 3973) RU/ml (p<0.05 from baseline).

**Table 1:** Biochemical data in 11 dialysis patients before and after 8 months of therapy with doxercalciferol.

	Pre-treatment	Post-treatment
Calcium (mg/dl)	8.6 ± 0.2	8.8 ± 0.2
Phosphorus (mg/dl)	6.3 ± 0.4	6.0 ± 0.4
Alkaline phosphatase (IU/l)	298 (146, 488)	175 (124, 414)
25(OH)vitamin D (ng/ml)	25.6 ± 2.7	
PTH (pg/ml)	517 (378, 1048)	559 (375, 922)
FGF23 (RU/ml)	705 (318, 1196)	1696 (565, 3973)*

The asterisk indicates a significant (p<0.05) change from baseline.

**Fig 1:**
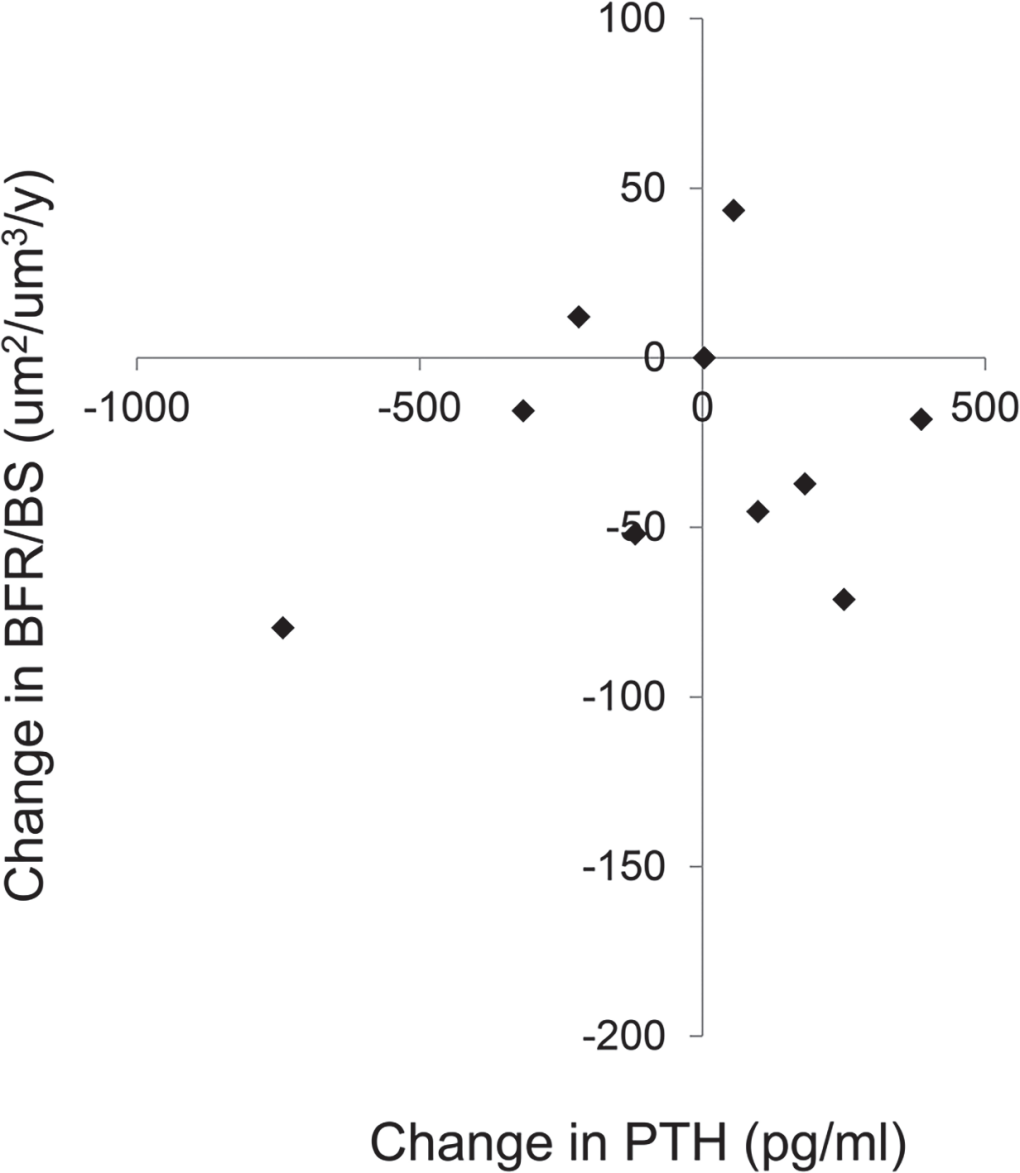
Changes in bone formation rate (BFR/BS) as a function of changes in circulating PTH concentrations.

At baseline, all subjects had evidence of secondary hyperparathyroidism and 6 of the 11 had evidence of defective mineralization, as defined by an abnormal osteoid volume combined with delays in osteoid maturation time. With therapy, osteoid volume, thickness, and surface decreased; indeed, while 73% of subjects had increased osteoid volume and 78% had increased osteoid maturation times at baseline, osteoid volume was normal in 82% of subjects after therapy, although 80% of subjects continued to demonstrate prolonged osteoid maturation times. Bone formation rate and bone volume did not significantly change with therapy. Values for bone histomorphometric variables are displayed in **Table 2**. Changes in bone formation rate, relative to changes in plasma PTH concentrations, are shown in **Fig. 1**; consistent with previous reports [24–26], changes in PTH were poor predictors of changes in bone turnover during therapy.

**Table 2:** Bone histomorphometry in 11 dialysis patients before and after 8 months of therapy with doxercalciferol.

	Pre-treatment	Post-treatment	Normal Range
**Turnover**			
Bone formation rate (BFR/BS) (um^2^/mm^3^/y)	50.0 ± 10.4	30.4 ± 7.5	8.0–73.4
**Mineralization**			
Osteoid volume (OV/BV) (%)	7.0 ± 1.1	4.4 ± 0.8*	0.2–5.8
Osteoid thickness (O.Th) (um)	12.7 ± 0.9	10.2 ± 0.6*	2.0–13.2
Osteoid surface (OS/BS) (%)	41.1 ± 3.9	31.7 ± 3.8*	4.3–37.0
Osteoid maturation time (OMT) (d)	15.3 ± 1.4	14.6 ± 2.1	1.2–11.5
Mineralization lag time (MLT) (d)	27.5 (23.7, 47.1)	35.8 (30.3, 46.4)	2.3–63.8
**Bone Volume**			
Bone volume (BV/TV) (%)	37.2 ± 3.7	36.1 ± 2.7	8.9–34.4
Trabecular thickness (Tb.Th) (um)	173 ± 19	159 ± 6	91–175
Trabecular separation (Tb.Sp) (um)	289 ± 18	307 ± 44	351–737

The asterisk indicates a significant (p<0.05) change from baseline.

* p<0.05 from baseline.

### Osteocyte Protein and RNA Expression at Baseline and After 8 Months of Therapy

**Fig 2:**
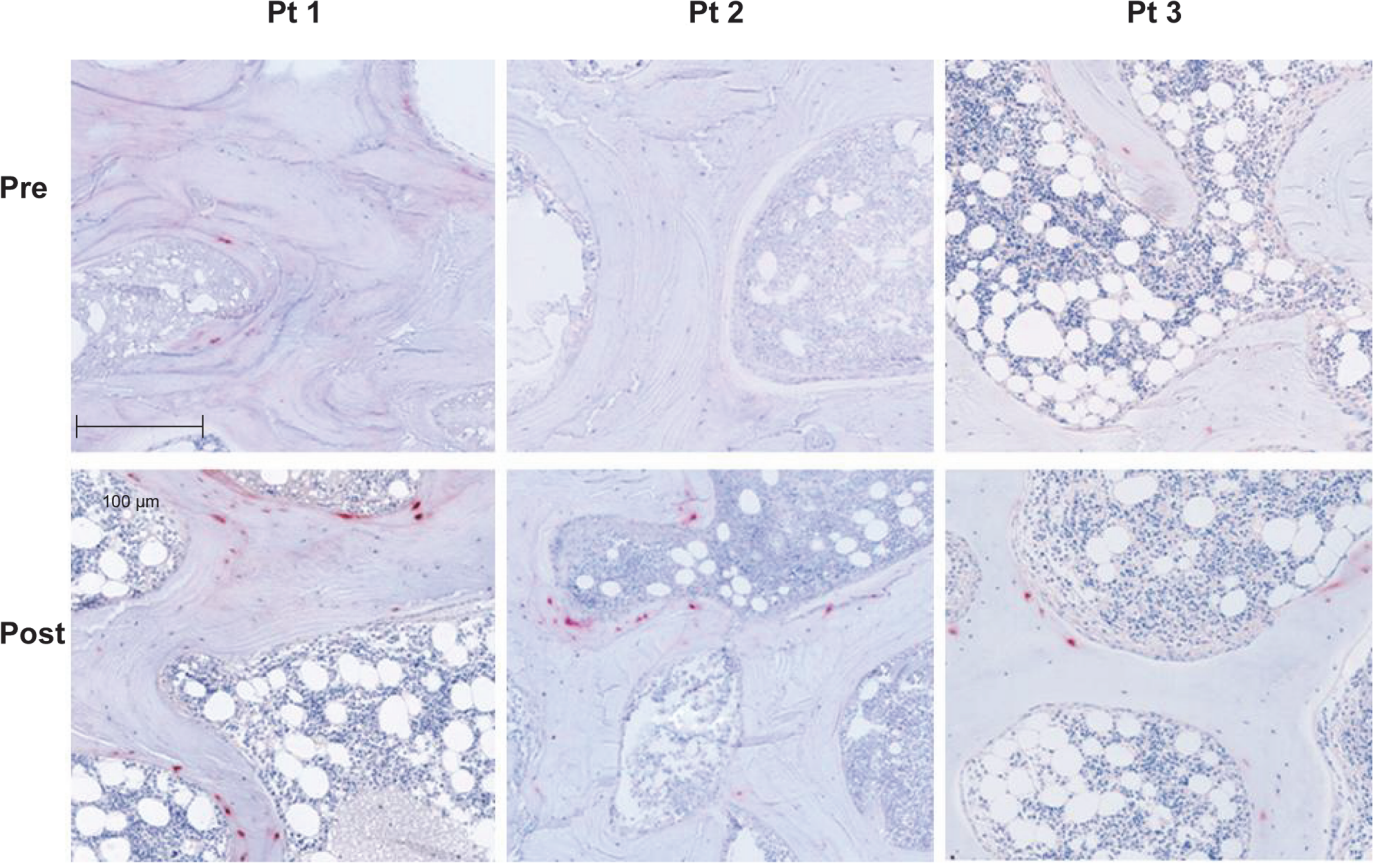
Immunohistochemical detection of FGF23 protein expression in 3 separate dialysis patients before (pre) and after 8 months of therapy with doxercalciferol. The bar indicates the length of a 100 μm segment of bone. (Magnification: 10x)

**Fig 3:**
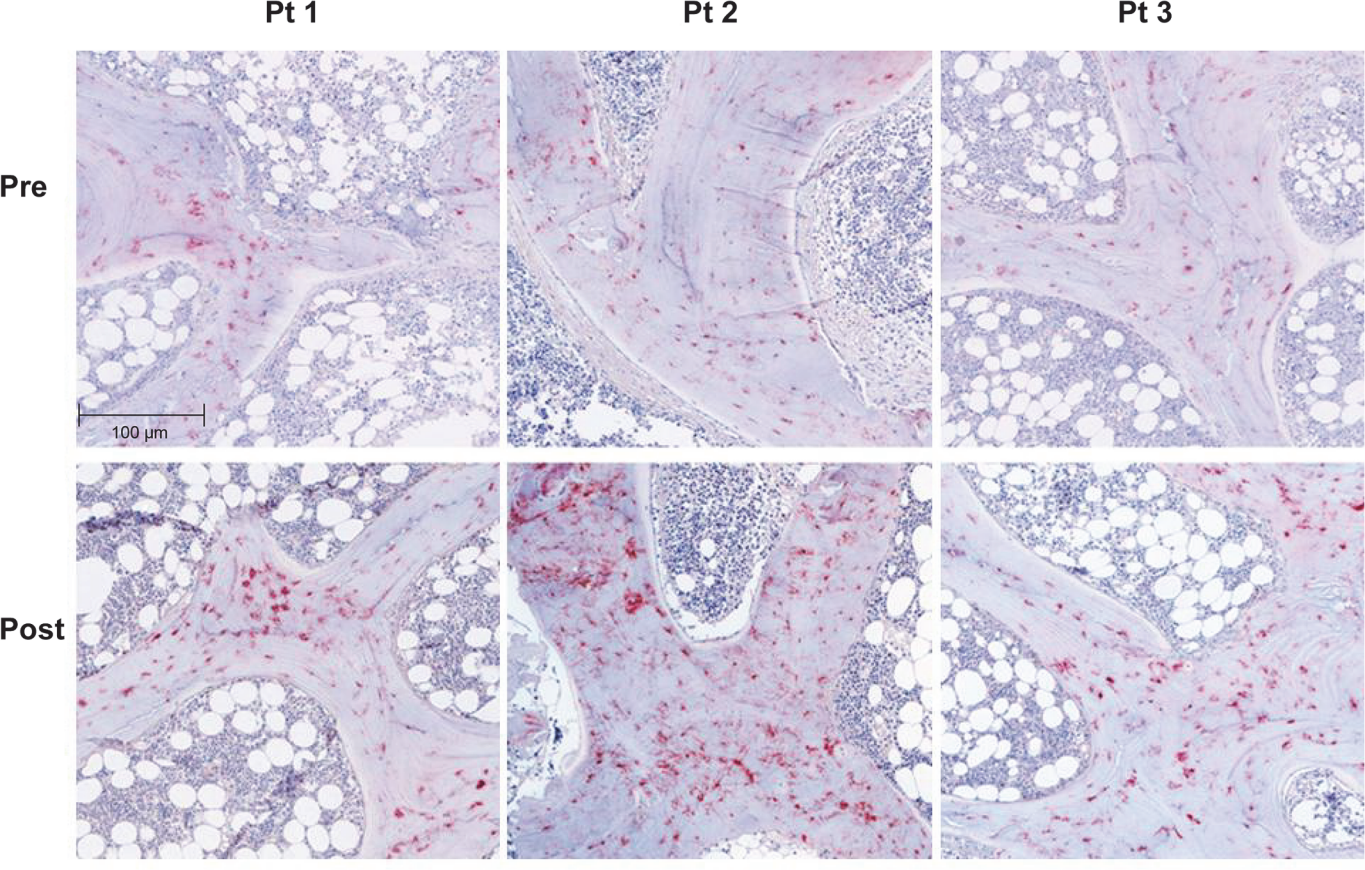
Immunohistochemical detection of DMP1 protein expression in 3 separate dialysis patients before (pre) and after (post) 8 months of therapy with doxercalciferol. The bar indicates the length of a 100 μm segment of bone. (Magnification: 10x)

**Fig 4:**
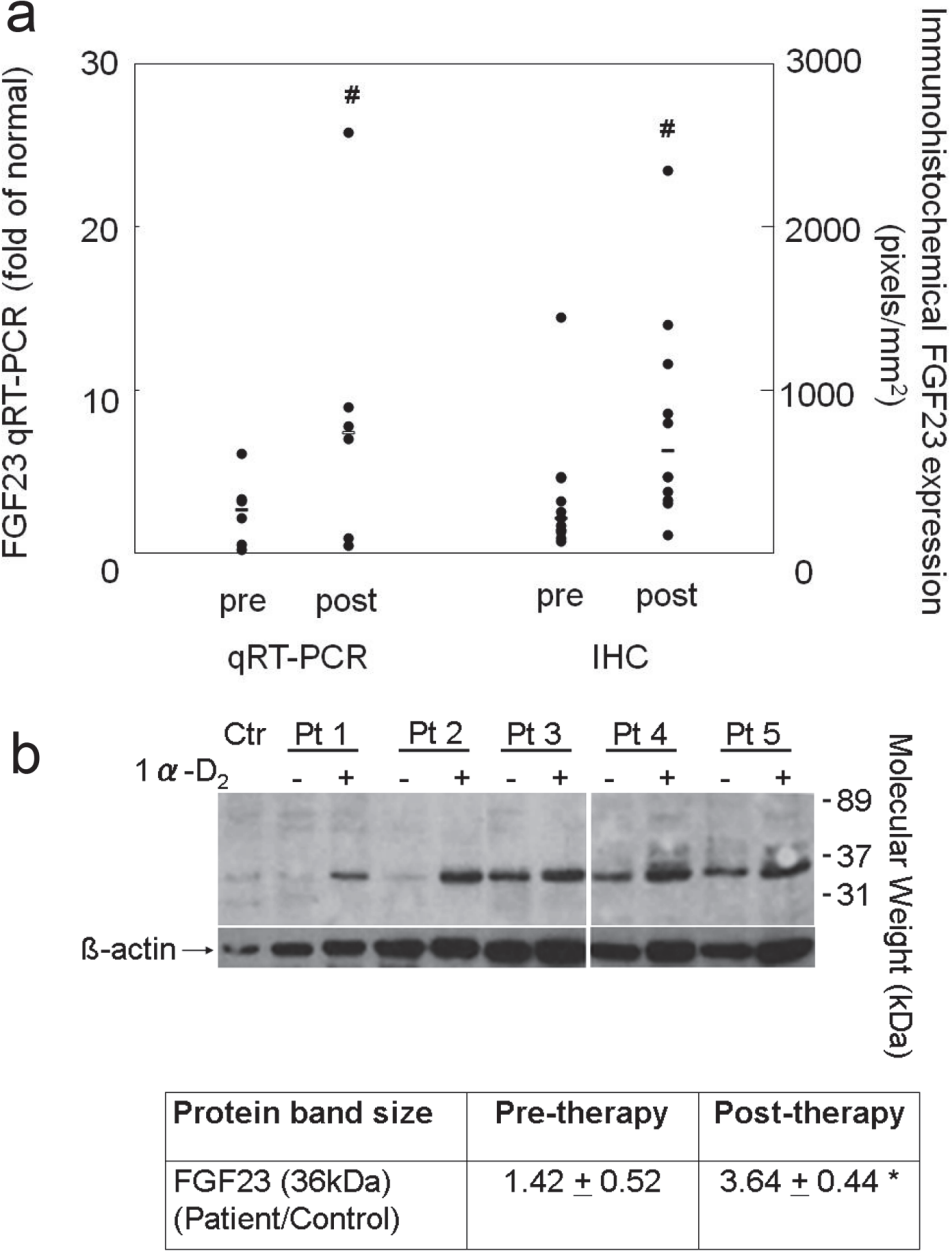
Changes in bone FGF23 expression with doxercalciferol therapy. a) The quantification of FGF23 mRNA (qRT-PCR) transcript and FGF23 protein as detected by immunohistochemistry (IHC). For qRT-PCR, gene expression in dialysis patients before and after doxercalciferol therapy was expressed relative to normal control and was calculated as 2^(ΔCt FGF23)/2^ (ΔCt GAPDH). Individual data points are shown; the bar indicates the median value. The number sign indicates a significant (p<0.05) change from baseline. b) Western blot analysis of bone extract for FGF23 demonstrated only the presence of full-length (36 kDa) FGF23 in 5 separate dialysis patients and an increase in full-length FGF23 expression with doxercalciferol. Abbreviations: Ctr: healthy control; Pt: patient. The asterisk indicates a significant (p<0.05) change from baseline

FGF23 and DMP1 were expressed primarily in trabecular bone (**Figs. 2 and 3**). DMP1 was expressed in osteocyte cell bodies and dendrites throughout bone while FGF23 was expressed in only a subset of osteocytes at the periphery of bone and only in osteocyte cell bodies. Bone FGF23 expression (**Fig. 2**), as determined by quantification of immunohistochemical staining, increased from baseline by 333 ± 139% (p<0.05 from baseline) (**Fig. 4A**) and FGF23 expression in bone correlated with circulating values of the protein (r = 0.46, p<0.05). After correcting for protein loading, Western blot analysis of 5 individuals, 2 of whom (Pts 1 and 2) displayed a large increase in immunohistochemical detection of FGF23 and 3 (Pts 3, 4, and 5) with smaller increases in FGF23 immunoreactivity, confirmed an overall increase in FGF23 protein after doxercalciferol therapy (p<0.05) (**Fig. 4B**) and also revealed that all of the immunoreactive FGF23 was full-length without evidence of fragments. An increase in FGF23 expression from first to second biopsy was confirmed by qRT-PCR (**Fig. 4A**).

**Fig 5:**
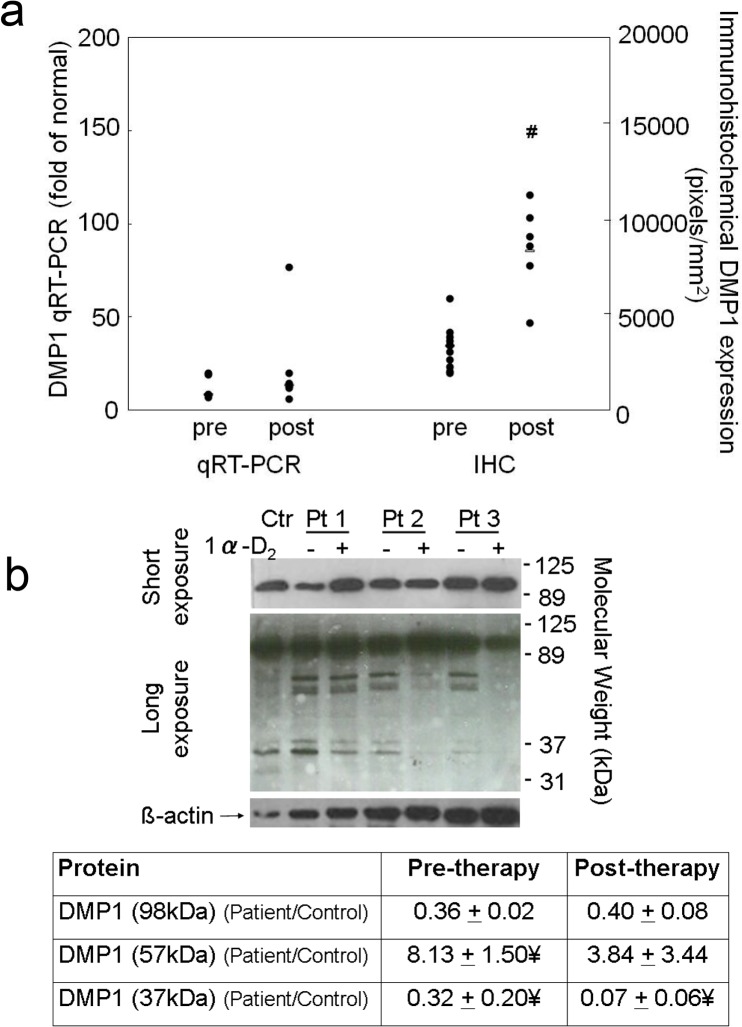
Changes in bone DMP1 expression with doxercalciferol therapy. a) The quantification of DMP1 mRNA (qRT-PCR) transcript and DMP1 protein as detected by immunohistochemistry (IHC). For qRT-PCR, gene expression in dialysis patients before and after doxercalciferol therapy was expressed relative to normal control and was calculated as 2^(ΔCt DMP1)/2^ (ΔCt GAPDH). Individual data points are shown; the bar indicates the median value. The number sign indicates a significant (p<0.05) change from baseline. b) Western blot analysis of bone extract for DMP1 demonstrated higher amounts of 57kDa and lower amounts of 37kDa fragments in bone extracts of dialysis patients than of healthy control. The presence of the 57kDa fragment did not differ from control after treatment with doxercalciferol. Pts 1, 2, and 3 correspond to Pts 1, 2, and 3 in Fig. 3. Abbreviations: Ctr: healthy control; Pt: patient; ¥: p<0.05 from healthy control

**Fig 6:**
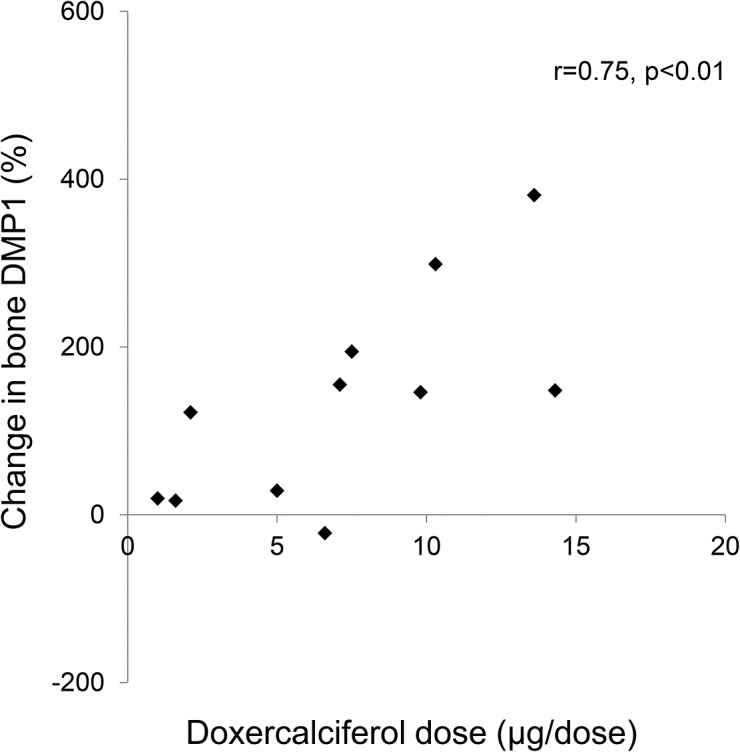
Correlation between changes in bone DMP1 expression and prescribed doxercalciferol dose.

**Fig 7:**
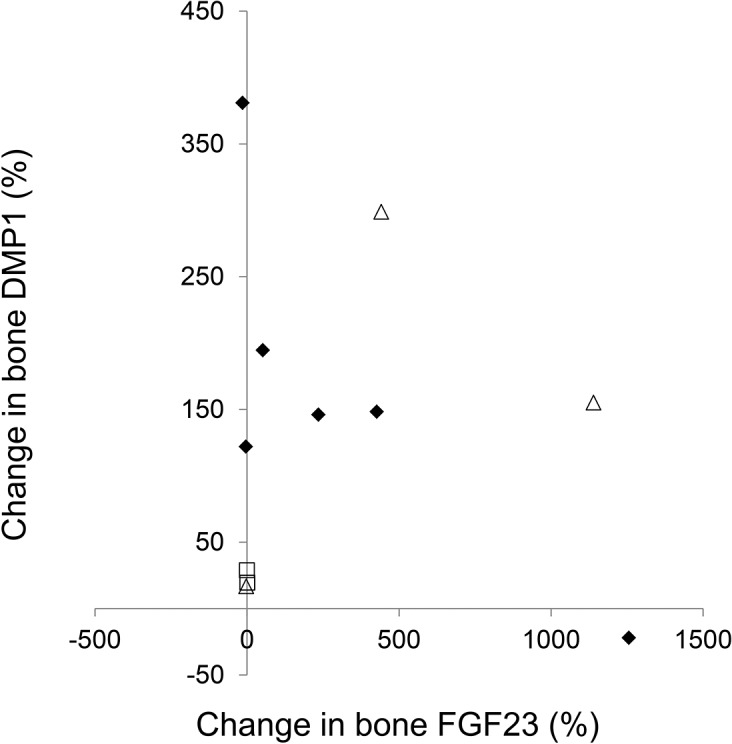
Changes in bone DMP1 expression as a function of changes in bone FGF23 expression. The three patients chosen for DMP1 Western blot analysis (Fig. 5B) are depicted by open triangles; the 5 patients chosen for FGF23 Western blot analysis (Fig. 3B) are depicted by open triangles and open squares.

Immunohistochemical analysis also revealed an increase in bone DMP1 protein expression (**Figs. 3 and 5**) of 133 ± 38% (p<0.01) from baseline. This increase in DMP1 protein correlated both with doxercalciferol dose (r = 0.75, p<0.01) (**Fig. 6**) and with change in circulating calcitriol concentration during the course of the study (r = 0.63, p<0.05). Interestingly, although both FGF23 and DMP1 increased with therapy, there was a high level of inter-patient variability in the magnitude of this response (**Fig. 7**). As determined by Western blot analysis of 3 patients (Pts 1, 2, and 3 displayed in the FGF23 blot), the majority of DMP1 was 98 kDa in size. Small amounts of 57 kDa and 37 kDa fragments were also present; greater amounts of the 57 kDa fragment were apparent in dialysis patients than in healthy controls and the quantity of both fragments decreased during doxercalciferol therapy (**Fig. 5B**). Changes in DMP1 RNA expression from 6 pairs of bone biopsies failed to reach statistical significance (**Fig. 5A**). Although both BMP1 and MMP-2 were increased in dialysis patients compared to control (9.1 ± 2.0 and 13.0 ± 2.3 fold increase from controls (p<0.05 for each enzyme, respectively)), RNA expression of each enzyme remained unchanged by doxercalciferol therapy (12.0 ± 3.5 and 18.3 ± 7.4 fold of control, respectively (p<0.05 from normal, NS from baseline)).

**Fig 8:**
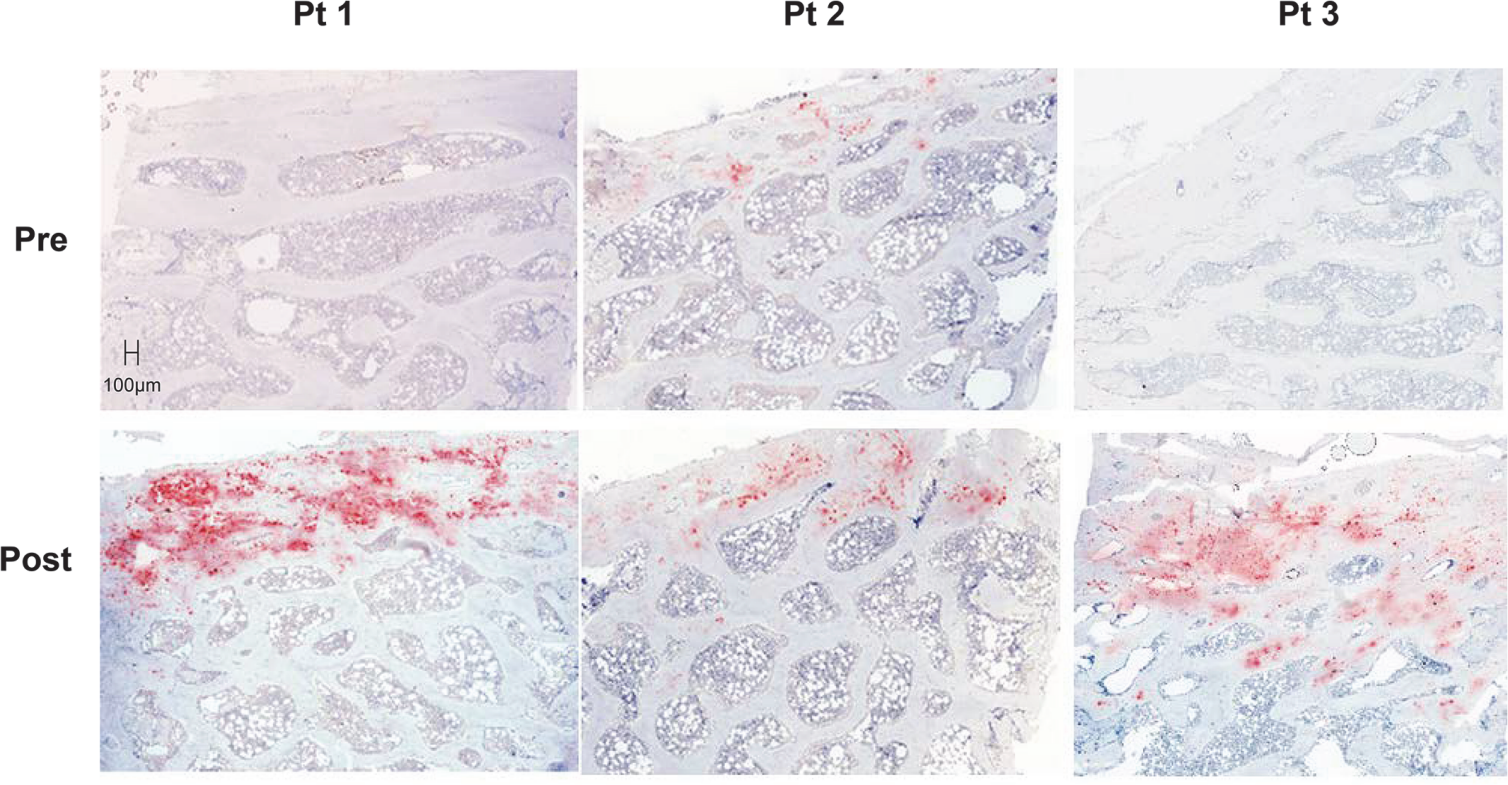
Immunohistochemical detection of sclerostin protein expression in 3 separate dialysis patients before (pre) and after (post) 8 months of therapy with doxercalciferol. The bar indicates the length of a 100 μm segment of bone. (Magnification: 10x)

**Fig 9:**
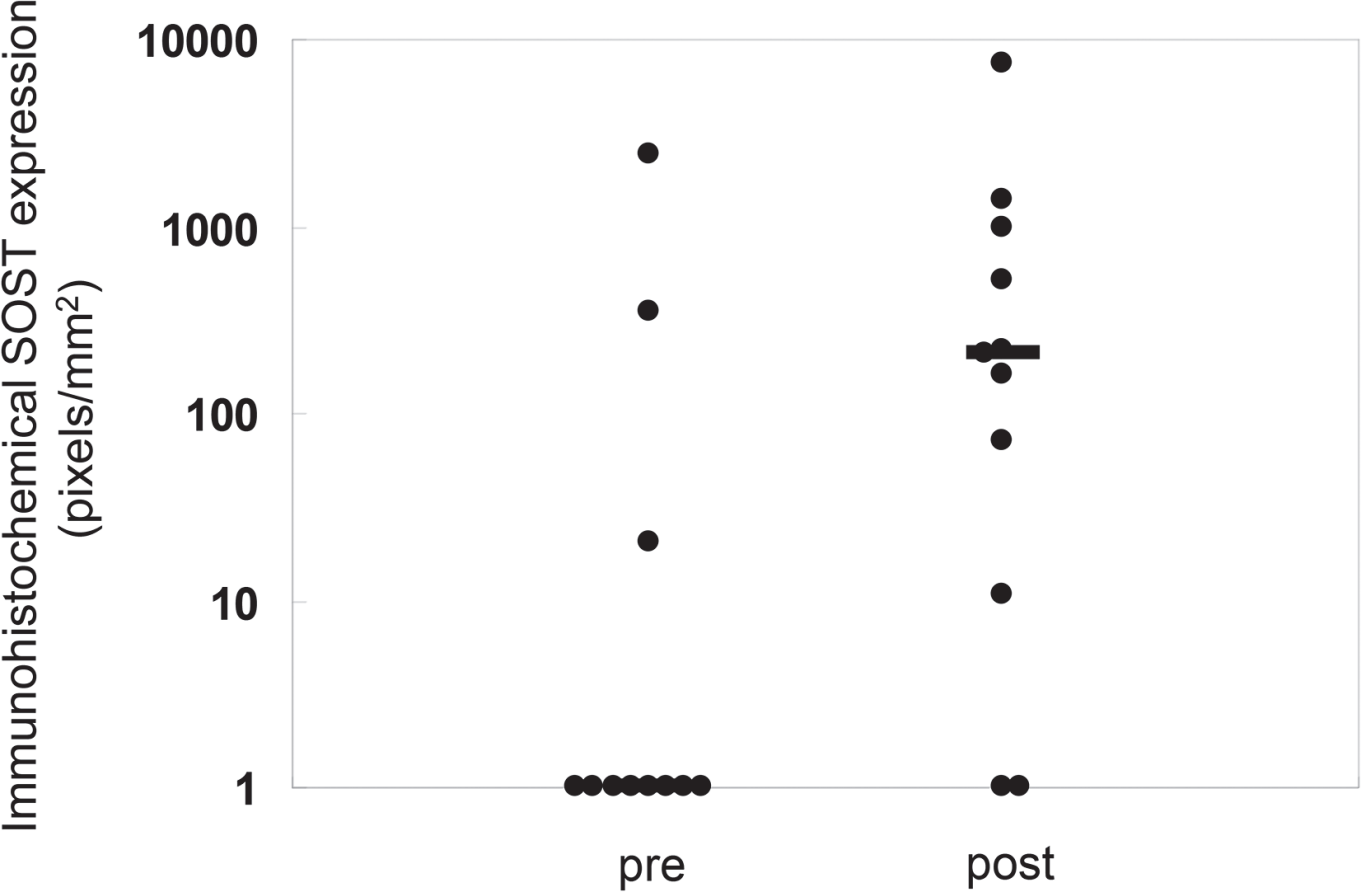
Quantification of sclerostin protein as detected by immunohistochemistry before and after doxercalciferol therapy. Individual data points are shown; the bar indicates the median value. The number sign indicates a significant (p<0.05) change from baseline.

**Fig 10:**
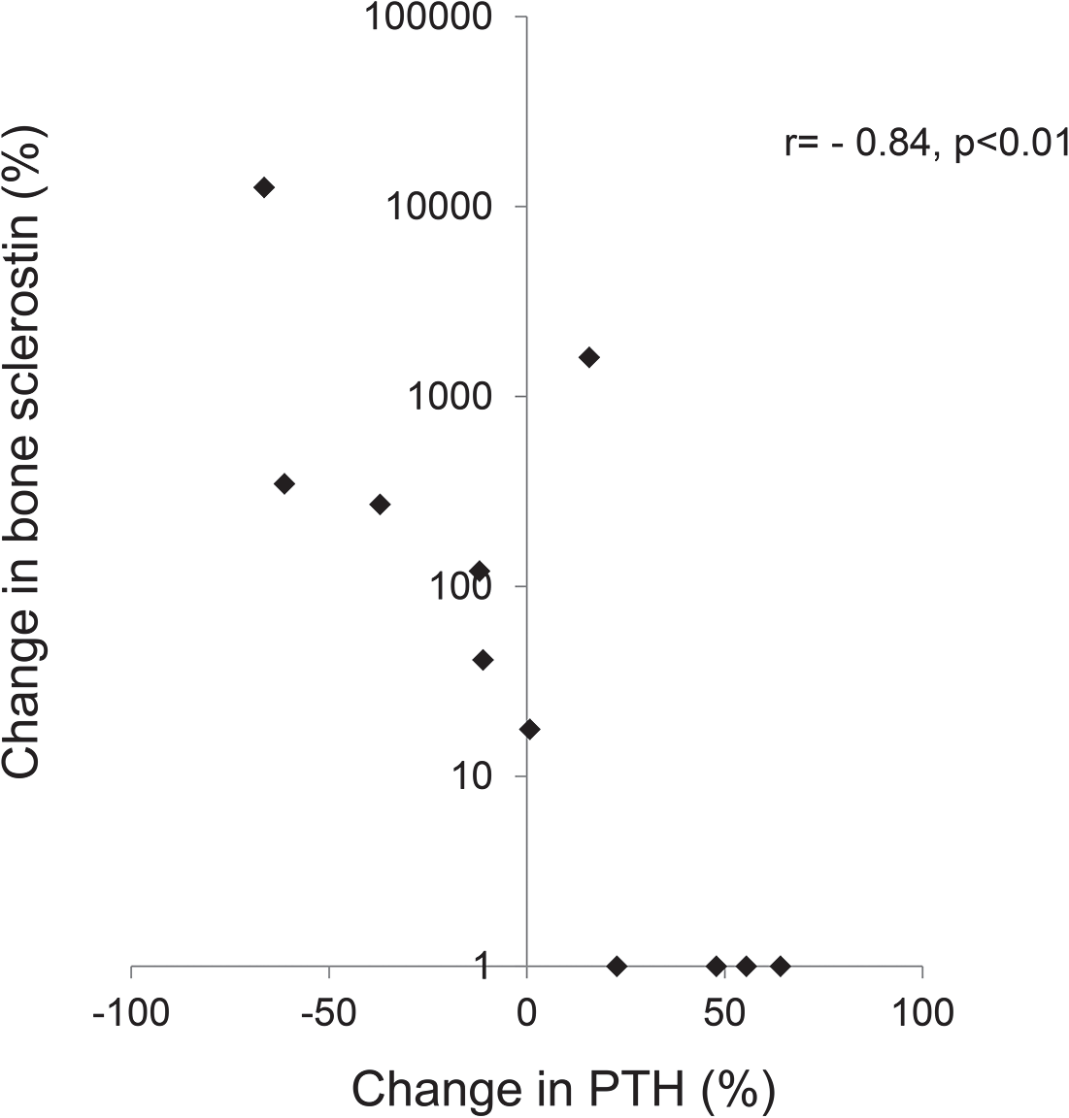
Change in bone sclerostin as a function of changes in plasma PTH concentration.

The vast majority of sclerostin expression was found in cortical bone in all patients. In those subjects with some positive trabecular staining for sclerostin, all positive staining was noted in peripheral trabeculae and cortical sclerostin expression was maximal in these individuals. Overall, both cortical and trabecular sclerostin expression were increased from baseline on the follow up bone biopsy (**Figs. 8 and 9**). Changes in circulating PTH concentration correlated with changes in bone sclerostin (r = − 0.84, p<0.01) (**Fig. 10**); notably, however, cortical sclerostin increased in 2 individuals in whom circulating PTH concentrations did not decrease. In the two individuals with no noted sclerostin increase in response to therapy, cortical sclerostin expression was completely absent both at baseline and PTH values did not decline from baseline in these individuals.

## Discussion

The current study demonstrates that changes in osteocytic protein expression occur with doxercalciferol therapy in pediatric dialysis patients and that these changes are readily apparent even with a small sample size. Bone FGF23 protein increased; this increase was due to an increase in the full-length molecule and no fragments were apparent. Bone DMP1, as detected by immunohistochemistry, also increased. DMP1 was present primarily in its full-length form with only very small amounts of 57kDa and 37kDa fragments present in healthy controls. Greater amounts of the 57kDa fragment were apparent in dialysis patients although the quantity of this fragment decreased with therapy. Sclerostin expression was apparent primarily in cortical bone and its expression increased with the administration of doxercalciferol.

Previous work in animals and humans has implicated FGF23 and DMP1 in skeletal mineralization. A complete lack of DMP1 in the context of normal renal function results in increased circulating FGF23, hypophosphatemia, low 1,25(OH)_2_vitamin D levels and diffuse osteomalacia/rickets. This phenotype is similar to that observed in animals and humans with primary increases in FGF23,[27–29] and transgenic expression of either the full-length DMP1 or the C-terminal 57kDa DMP1 fragment reverses the excess FGF23 and osteomalacia present in the DMP1 null mice[9], suggesting that DMP1 acts to downregulate FGF23 expression. Our previous work demonstrated that both bone FGF23 and DMP1 expression are elevated in CKD patients; the expression of these proteins correlate directly with each other and higher expression of both proteins is associated with decreased osteoid accumulation (i.e. improved mineralization).[2] The finding that higher expression of FGF23 and DMP1 is related to improved indices of skeletal mineralization appeared contradictory to data from animals and humans with normal kidney function, leading us to postulate that although DMP1 expression is upregulated in CKD, its function is likely impaired.

By immunohistochemistry, both FGF23 and DMP1 protein appeared to increase in bone in response to doxercalciferol therapy. In both normal controls and in dialysis patients, bone FGF23 appeared to be almost exclusively in its full-length form. Although it is possible that a small amount of fragments, not detected by Western blot analysis, is produced in bone, the current study is consistent with data suggesting that the majority of FGF23 in circulation dialysis patients is in its intact and biologically active form. The lack of fragments in the healthy control further suggests that the majority of cleavage of FGF23 in healthy individuals[30] occurs after secretion from bone by systemic enzymes, such as furin.[31] The antibody used for detection of DMP1 by immunohistochemistry and for Western blot binds both the full-length (98 KDa) and the C-terminal (57 kDa) moieties. Thus, the immunohistochemical results likely reflect the effect of doxercalciferol on both of these moieties combined. Using this same antibody, the Western blot analysis revealed that DMP1 was present in primarily in the full-length (98 kDa) form in the bone of both healthy control and in all dialysis patients. This finding contrasts with previous data in mice suggesting that the vast majority of DMP1 undergoes post-translational cleavage, leaving less than 1% of the protein in the full-length form[32] and suggests that DMP1 biology in humans may differ from that in mice.[32] Moreover, the finding that both the DMP1 transcript and protein increase in dialysis patients in response to doxercalciferol differs from previous data from Nociti and colleagues in which vitamin D repressed DMP1 mRNA expression in murine cementoblasts and osteocytes *in vitro* [33]. The discrepancy between the two studies may be attributable to differences between species, to differences between osteocyte biology in the context of normal kidney function versus kidney disease, or to differences between the effects of vitamin D on DMP1 transcription and translation. Previous reports have suggested that MMP2 and BMP1 cleave DMP1,[34, 35] and, although their expression could not be evaluated by immunohistochemistry or by Western blot, RNA of both appeared to be increased from normal. However, therapy did not alter their RNA expression, suggesting that changes in DMP1 cleavage were not due to decreased transcription of these enzymes.

Although the majority of the DMP1 was present as a 98 kDa moiety, DMP1 fragments were also readily detected in the bone extracts from humans in this study. In the dialysis patients, the intensity of the DMP1 bands (normalized by the intensity of the ß-actin bands to control for protein loading and normalized by quantities in the healthy control) revealed a greater quantity of the 57 kDa DMP1 fragment in dialysis patients at baseline than in controls. The quantity of this fragment appeared to decrease in response to treatment, while the quantity of the full-length, 98 kDa DMP1 moiety, increased. Thus, the overall increase in DMP1 seen on immunohistochemistry primarily reflected a change in the 98 kDa DMP1 moiety. Whether suppression of the 57 kDa fragment in response to doxercalciferol therapy affects osteocyte function and bone biology in humans is as yet unclear, but warrants further investigation, particularly in light of murine data suggesting that the 57 kDa DMP1 fragment potentiates mineralization and osteocyte maturation.[36, 37] Furthermore, Martin et al.[9] recently demonstrated that the 57 kDa DMP1 fragment suppresses FGF23 in the presence of phosphate regulating endopeptidase homologue, X-linked (PHEX) but stimulates FGF23 in its absence.[9] Although, due to the bone fixation conditions, PHEX activity could not be assessed by either immunohistochemistry or Western blot in the current study, differences in expression of this enzyme might account for the marked inter-patient variability in FGF23 and DMP1 in response to doxercalciferol and also warrants further investigation.

Although sclerostin, an inhibitor of *Wnt* signaling in osteocytes,[7] is believed to be suppressed by PTH and has been proposed to mediate changes in skeletal FGF23,[6] the current study calls this paradigm into question. Changes in sclerostin correlated with changes in PTH values; however, an increase in bone sclerostin expression was found in some patients in whom circulating PTH values did not change at all. It is interesting to note that, although not statistically significant due to the relative small number of study participants, bone turnover tended to decrease in this cohort and values for bone formation rate were unrelated to circulating PTH values. This finding confirms previous data demonstrating that a wide range of PTH values are associated with normal rates of bone turnover[38], particularly in patients treated with high doses of vitamin D analogues[13] and that PTH levels poorly reflect bone biology. The current data—i.e. that bone sclerostin increases in response to doxercalciferol therapy, even in the absence of a decrease in PTH—suggests that changes in osteocyte biology may play a part in mediating skeletal resistance to PTH.

The current study yields insights into the response of different osteocytic proteins to doxercalciferol. It is important to consider, however, that this study was performed in pediatric patients who were dialyzed exclusively with CCPD and who were primarily Hispanic in ethnicity. Some studies have suggested that circulating FGF23 values may differ according to patient ethnicity;[39]whether this difference translates into differences in osteocytic protein production and cleavage is unknown but is possible. Moreover, differences in patterns of sclerostin expression have been noted between adults and children [7] and warrants further evaluation.

In conclusion, despite greater than normal DMP1 expression in patients with advanced CKD, this protein appears to be abnormally cleaved in dialysis patients. FGF23 expression appears in its full-length, uncleaved form in bone of dialysis patients. Therapy with doxercalciferol increases expression of full-length FGF23 as well as total DMP1 and decreases the presence of the 57 kDa DMP1 fragment in the bone of pediatric dialysis patients with secondary hyperparathyroidism. Increases in sclerostin expression occurred as a result of doxercalciferol therapy. The effect of these changes on long-term outcomes remain to be determined.
